# Molecular mechanisms of NMDA excitotoxicity in the retina

**DOI:** 10.1038/s41598-023-45855-0

**Published:** 2023-10-27

**Authors:** Galina Dvoriantchikova, Michelle Fleishaker, Dmitry Ivanov

**Affiliations:** 1 Department of Ophthalmology, Bascom Palmer Eye Institute, University of Miami Miller School of Medicine, 1638 NW 10Th Ave, Miami, FL 33136 USA; 2 Department of Microbiology and Immunology, University of Miami Miller School of Medicine, Miami, FL 33136 USA

**Keywords:** Neuroimmunology, Ion channels in the nervous system, Molecular neuroscience, Diseases of the nervous system, Neurodegeneration, Neuroscience, Visual system, Retina, Cell death and immune response, Tumour-necrosis factors, Immune cell death, Innate immunity, Pattern recognition receptors, Immunology, Neuroimmunology, Biological techniques, Sequencing, RNA sequencing, Eye diseases, Retinal diseases, Medical research, Experimental models of disease, Nervous system, Sensory systems, Visual system, Retina

## Abstract

NMDA excitotoxicity, as a part of glutamate excitotoxicity, has been proposed to contribute significantly to many retinal diseases. Therefore, understanding mechanisms of NMDA excitotoxicity will provide further insight into the mechanisms of many retinal diseases. To study mechanisms of NMDA excitotoxicity in vivo, we used an animal model in which NMDA (20 mM, 2 µL) was injected into the vitreous of mice. We also used high-throughput expression profiling, various animals with reduced expression of target genes, and animals treated with the oral iron chelator deferiprone. We found that the expression of many genes involved in inflammation, programmed cell death, free radical production, oxidative stress, and iron and calcium signaling was significantly increased 24 h after NMDA treatment. Meanwhile, decreased activity of the pro-inflammatory TNF signaling cascade and decreased levels of ferrous iron (Fe^2+^, required for free radical production) led to significant neuroprotection in NMDA-treated retinas. Since increased TNF signaling activity and high Fe^2+^ levels trigger regulated necrosis, which, in turn, lead to inflammation, we proposed an important role in NMDA excitotoxicity of a positive feedback loop in which regulated necrosis promotes inflammation, which subsequently triggers regulated necrosis.

## Introduction

The retina contains a variety of excitatory retinal neurons whose main neurotransmitter is glutamate^1^. Thus, it is not surprising that retinal damage due to disease (e.g., glaucoma and ischemic optic neuropathy) or injury results in the release of significant amounts of glutamate into the extracellular space^2–5^. Extracellular glutamate is not a passive witness to the developing pathology in the retina, as it plays a significant and active role. Glutamate neurotransmission is tightly regulated by an array of receptors and transporters to minimize the presence of excess glutamate in the extracellular space^6^. Meanwhile, high levels of extracellular glutamate lead to excitotoxicity, an important factor of many neurodegenerative diseases^6^. Within the retina, glutamate excitotoxicity can lead to a significant deterioration in vision and, in some cases, even blindness^2–6^. One of the mechanisms leading to glutamate excitotoxicity is NMDA receptor overactivation, which is known as NMDA excitotoxicity^6^. The objective of this study was to investigate mechanisms of NMDA excitotoxicity in the retina.

Retinal ganglion cells (RGCs) are the only retinal neurons that send their axons to the visual cortex of the brain^1^. RGC death due to disease or injury ultimately leads to blindness^2–6^. At the same time, amacrine cells modulate the signal transmitted from bipolar cells to RGCs within the inner plexiform layer and modulate the activity of RGCs within the ganglion cell layer^7^. High NMDA levels in the retina lead to the death of RGCs and amacrine cells^8–11^. However, the dynamics of the death of these neurons is different: amacrine cells begin to die first, while the death of RGCs is delayed^8–11^. It was also observed that murine amacrine cells die predominantly via necrosis during the first hour, while apoptosis of these cells is observed only by the third hour after NMDA treatment^8,9^. The death of RGCs in the presence of NMDA is a more complex problem. While the significant RGC death in the presence of NMDA is well documented in vivo, there is uncertainty as to whether NMDA leads to significant RGC death in vitro^8–12^. The results obtained in Dr. Barres’ laboratory suggest that NMDA is either non-toxic or even promotes the survival of RGCs in vitro^9,12^. Other studies suggest that NMDA causes RGC death in vitro^13^. However, even so, these neurons are probably less sensitive to NMDA excitotoxicity in vitro than other types of neurons^9,12,13^. Thus, it is not entirely clear whether RGC death in vivo is a result of overactivation of NMDA receptors or an indirect effect of NMDA excitotoxicity. The findings from this study suggest that NMDA-induced inflammation results in the activation of TNF signaling and an increase in ferrous (Fe^2+^) iron levels, leading to RGC death. Thus, the mechanism of NMDA excitotoxicity is more complex and is determined by the direct and indirect effects of NMDA on retinal neurons.

## Results

### The activity of retinal NMDA receptors leads to significant changes in gene expression accompanied by RGC death

**Figure 1:**
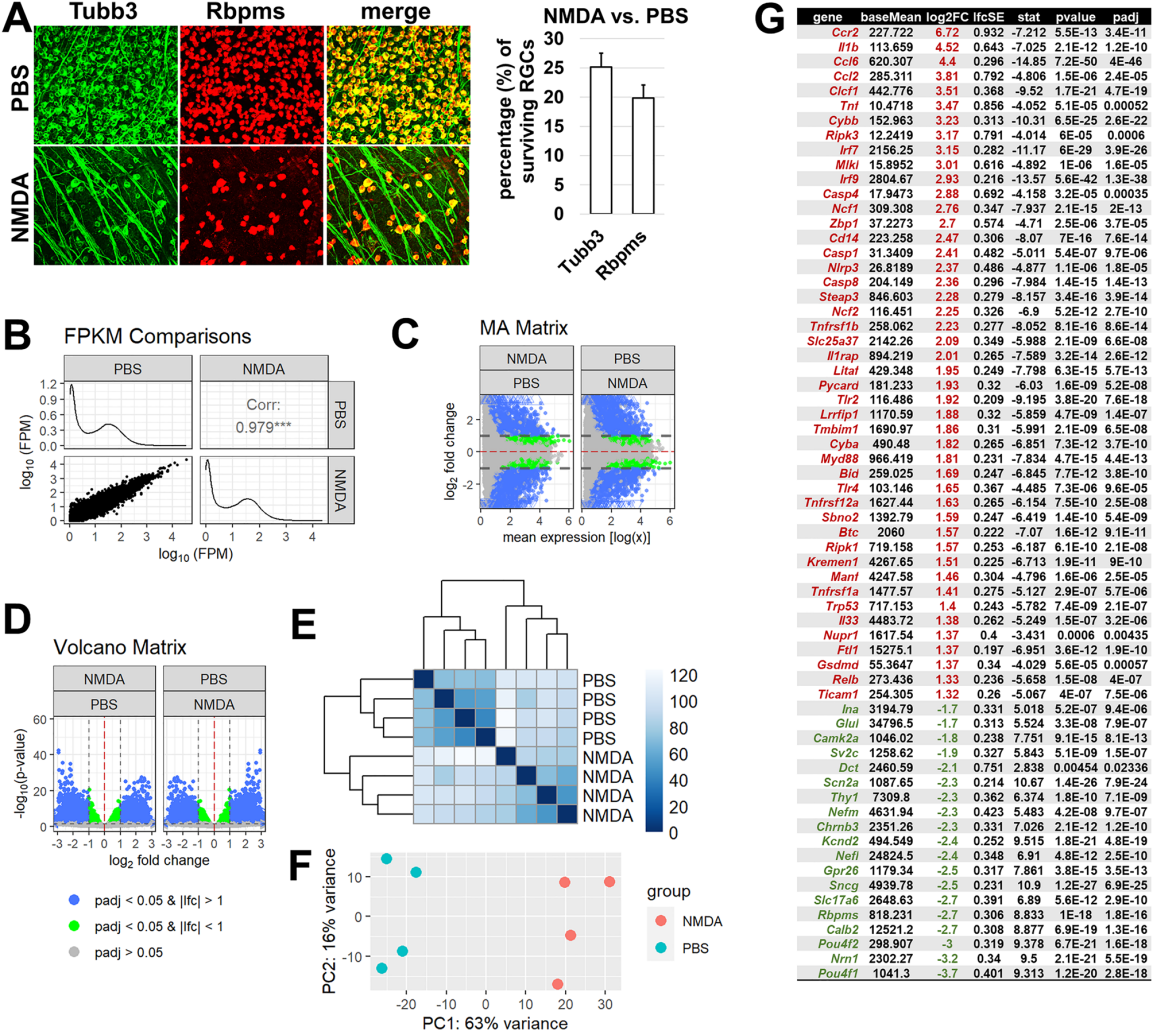
Retinal NMDA excitotoxicity is accompanied by significant changes in gene expression. (**A**) NMDA injection into the vitreous leads to significant neuronal death in the ganglion cell layer (GCL) after 7 days. (**B)** Correlation (Corr) value and FPKM distributions were generated to visualize the correlation between levels of gene expression in NMDA-treated and control (PBS-treated) retinas 24 h after treatment. (**C**,**D**) MA and volcano plots show dramatic and statistically significant changes in gene expression after NMDA treatment. padj is *p value* adjusted; lfc is the log2 fold change (Log2FC) between two conditions (NMDA vs. PBS). (**E**,**F**) A heatmap of the sample-to-sample distances (sample clustering, **E**) and the principal component analysis (PCA) plot of the samples (**F**) illustrate the significant difference between NMDA-treated and PBS-treated retinas. (**G**) The table provides examples of genes whose expression is increased (highlighted in dark red) and whose expression is decreased (highlighted in green) in the NMDA-treated retinas. Log2FC is the logarithm (log2) of the fold change in gene expression (NMDA vs. PBS). This is a standard approach for characterizing gene expression in bioinformatics. To get the usual value of changes in gene expression, this formula (2^Log2FC^) should be used.

RGCs and displaced amacrine cells are found only in the ganglion cell layer (GCL) of the retina in a relatively equal proportion^14^. In their paper published in 2004, Ullian et al. showed that RGCs are not sensitive to glutamate and NMDA excitotoxicity, while amacrine cells are very sensitive and die quickly in the presence of glutamate and NMDA^9^. Before and after this publication, there was much evidence that high levels of extracellular glutamate and NMDA are toxic to RGCs, leading to their death^2–6,8,10,11,13^. However, the presence of this publication has led to more careful consideration of which cell types in the retina are more sensitive to glutamate and NMDA excitotoxicity. To study the effects of extracellular NMDA on the survival of neurons in the GCL of the retina, we injected NMDA (20 mM, 2 µL; n = 6) and phosphate buffered saline (PBS as a control, 2 µL; n = 6) into the vitreous of the wild type (WT) mice. NMDA was injected into the left eyes of the animals and PBS was injected into the right eyes of the same animals. To determine the number of surviving neurons and RGCs in the GCL, the retinas of these animals were collected 7 days after treatment, and whole retina flat mounts were stained using a neuronal marker Tubb3 and a RGC marker Rbpms (Fig. 1A). The percentage of surviving cells was determined for each animal as the ratio of the mean number of cells counted in the NMDA-treated left eye to the mean number of cells counted in the PBS-treated right eye. The mean values obtained for a group of six animals for each of the studied cell markers are shown in Fig. 1A. We found that only 25 ± 2% (n = 6) of the Tubb3-positive GCL neurons and 19 ± 2% (n = 6) of the Rbpms-positive RGCs survived the NMDA treatment (Fig. 1A). Since Tubb3-positive GCL neurons correspond to the entire population of surviving neurons (RGCs and displaced amacrine cells), the percentage of surviving amacrine cells should be about 6%. This number of surviving amacrine cells is significantly less than the number of surviving RGCs, indicating that amacrine cells are more sensitive to NMDA excitotoxicity than RGCs.

To study the effects of NMDA excitotoxicity at the molecular level, we injected NMDA (20 mM, 2 µL; n = 4) and PBS (2 µL; n = 4) into the vitreous of the WT mice. The retinas of these animals were collected 24 h after treatment and used in RNA-seq analysis to examine changes in gene expression (Supplementary Fig. S1). To reach a significant depth of sequencing and thus obtain information on all types of retinal cells and not just on photoreceptors, which make up 70% of all retinal cells, we sequenced 52,309,779 ± 4,094,028 fragments (or more than 100 M reads) on average per library among which 40,441,184 ± 2,887,342 fragments were uniquely mapped to the mouse genome. The differential expression analysis of our RNA-seq data indicates that gene expression in NMDA-treated retinas is significantly altered compared to PBS-treated control retinas. This follows from the value of the correlation coefficient, MA and volcano plots, sample clustering, and principal component analysis (PCA) (Fig. 1B–F). The number of genes whose expression was statistically significantly (*p-value* adjusted [padj] < 0.05) increased by two or more times (log2 fold change [log2FC] ≥ 1) was 1422, while the number of genes whose expression was statistically significantly (padj < 0.05) reduced by two or more times (log2FC ≤ − 1) was 1352 (Fig. 1G, Supplementary Data S1). This data indicates that NMDA excitotoxicity has a significant impact on gene expression in the retina 24 h after treatment.

### NMDA excitotoxicity is accompanied by an increased expression of genes of signaling cascades, the high activity of which is dangerous for retinal neurons

**Figure 2:**
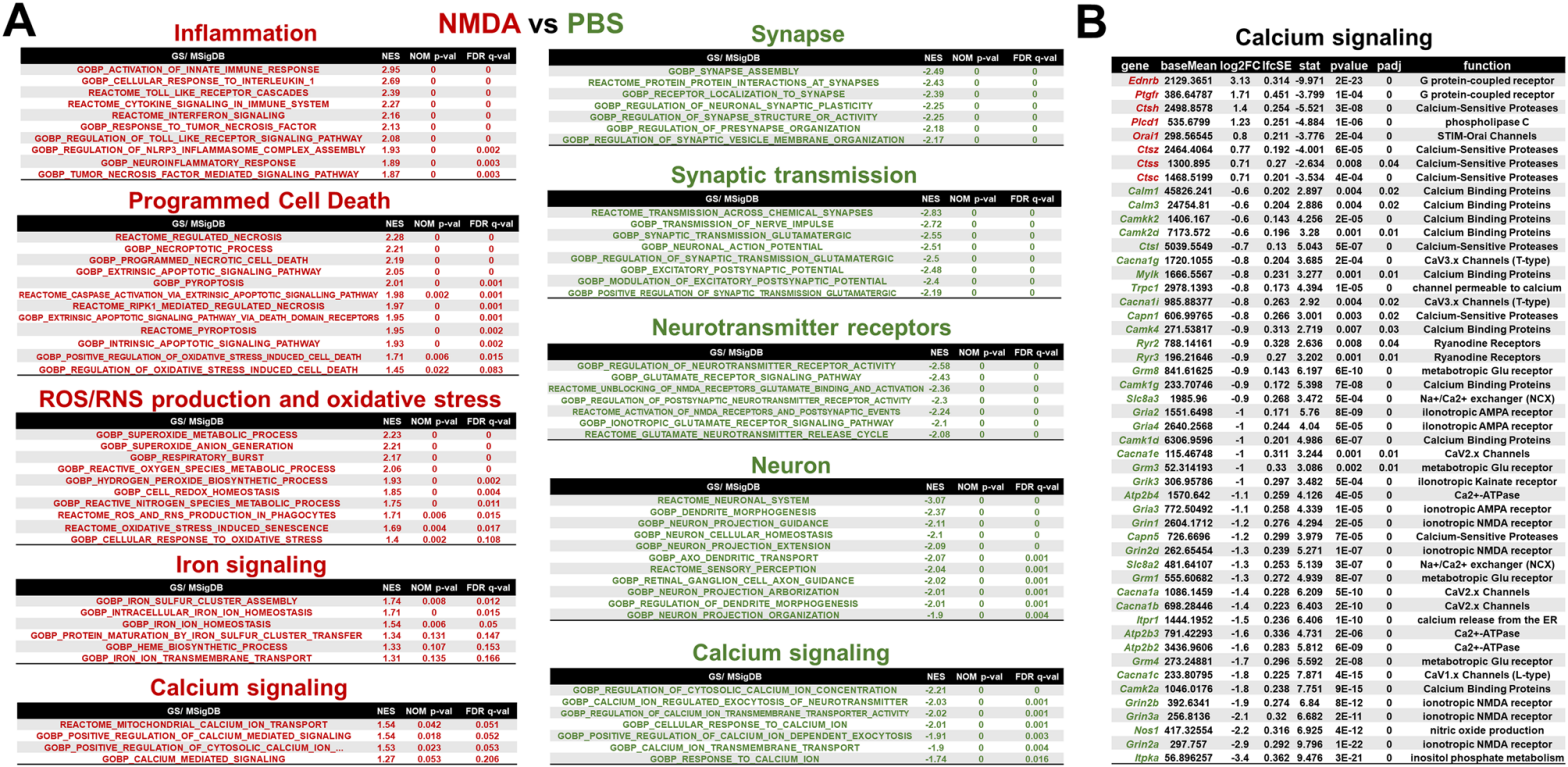
Gene expression analysis revealed a diversity of signaling cascades and biological processes triggered by NMDA in the retina. (**A**) NMDA increases the expression of genes involved in signaling cascades and biological processes whose activity can lead to retinal damage. At the same time, NMDA reduces the expression of genes whose activity is necessary for normal retinal function. (**B**) The table shows examples of genes involved in calcium signaling whose expression is not only increased but also decreased in NMDA-treated retinas.

Significant changes in the expression of many genes do not allow us to understand the impact of these changes until they are attributed to specific processes and signaling cascades. To this end, we used Gene Set Enrichment Analysis (GSEA), a powerful analytical method for interpreting RNA-seq data^15^. The results of the GSEA analysis indicated that most biological processes and signaling cascades whose gene expression is significantly upregulated 24 h after NMDA treatment fall into the following groups: (1) inflammation, (2) programmed cell death; (3) reactive oxygen and nitrogen species (ROS/RNS) production and oxidative stress, (4) iron signaling, and (5) calcium signaling (Fig. 2A, Supplementary Data S2). The biological processes and signaling cascades presented in the first (1) group indicate the important role of the innate immune system in general and such cascades as TNF signaling, IL1b signaling, toll like receptor (TLR) signaling, and interferon signaling, in particular (Fig. 2A). The second (2) group includes not only apoptosis but also regulated necrosis (e.g., necroptosis and pyroptosis). The connection of this group with the third (3) group should also be noted (GOBP Positive Regulation of Oxidative Stress Induced Cell Death [FDR q-val = 0.015], GOBP Regulation of Oxidative Stress Induced Cell Death [FDR q-val = 0.083], Fig. 2A). The third (3) group reflects an increased expression of genes, the activity of which may lead to a significant production of ROS/RNS with subsequent oxidative stress. We note the dependence of the third (3) group on the fourth (4) group, since the increased activity of biological processes and signaling cascades belonging to the fourth (4) group may lead to ferrous (Fe^2+^) iron accumulation. Ferrous (Fe^2+^) iron is a catalyst for the Fenton/Haber–Weiss reaction leading to the production of large amounts of free radicals (ROS/RNS). This suggests that oxytosis/ferroptosis, a type of regulated necrosis dependent on high free radical and ferrous (Fe^2+^) iron levels, may also be involved in NMDA excitotoxicity.

While biological processes and signaling cascades whose genes are upregulated after NMDA treatment are quite diverse, a significant number of biological processes and signaling cascades whose genes are downregulated 24 h after NMDA treatment are solely responsible for maintaining normal neuronal function (Fig. 2A). Since NMDA receptor activity leads to enhanced Ca^2+^ entry into neurons, it was not surprising that NMDA treatment affected the activity of the corresponding biological processes and signaling cascades (Fig. 2). However, it was surprising that some of their genes were upregulated while others were downregulated (Fig. 2). It should be noted that the expression of many NMDA receptors was significantly reduced (*Grin2a*, log2FC = − 2.9, padj = 0; *Grin3a*, log2FC = − 2.1, padj = 0; *Grin2b*, log2FC = −1.9, padj = 0; etc., Fig. 2B, Supplementary Data S1). This may be due to the attempts of neurons to compensate for the high content of extracellular NMDA. Another explanation for this fact may be the death of neurons, which leads to a decrease in the transcripts corresponding to them. This could also explain the reduced expression of genes responsible for maintaining normal neuronal function (Fig. 2A, Supplementary Data S2). An additional confirmation of this may be a reduced expression of markers of retinal neurons (*Pou4f1* [Brn3a], log2FC = − 3.7, padj = 2.8·10^–18^; *Pou4f2* [Brn3b], log2FC = − 3, padj = 1.6·10^–18^; *Rbpms*, log2FC = − 2.7, padj = 1.8·10^–16^; *Nefl*, log2FC = − 2.4, padj = 2.5·10^–10^; *Thy1*, log2FC = − 2.3, padj = 7.1·10^–9^; etc.). Thus, the totality of our data suggests that NMDA-induced inflammation, free radical production, and oxidative stress are accompanied by the death of retinal neurons through apoptosis and regulated necrosis.

### Decreased activity of signaling cascades controlling inflammation and regulated necrosis alleviates NMDA excitotoxicity

**Figure 3:**
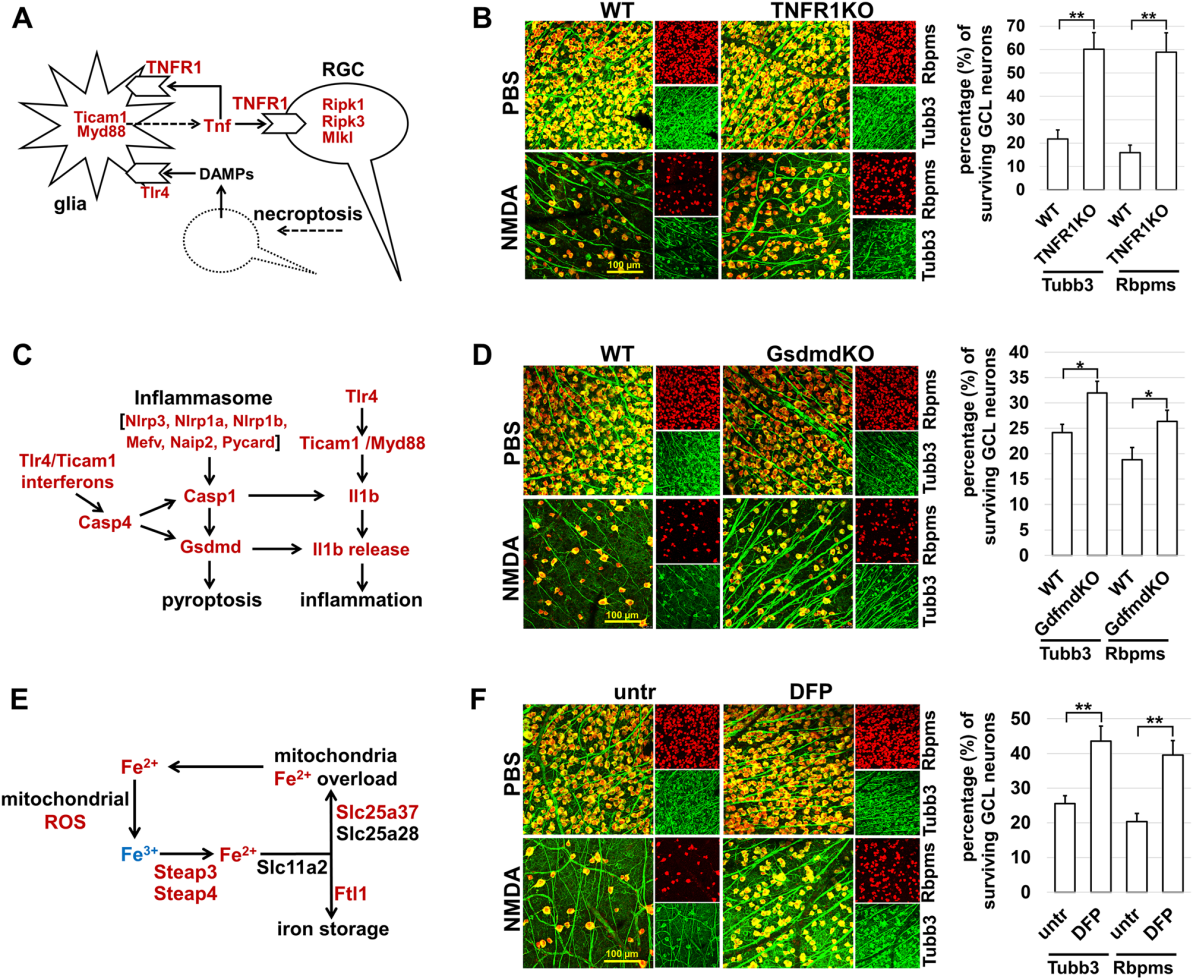
Signaling cascades controlling inflammation and regulated necrosis contribute significantly to NMDA excitotoxicity. (**A**) While the Tnf cytokine triggers an inflammatory response by activating TNFR1 receptors on the surface of glial cells (astrocytes and microglia), it can trigger RGC regulated necrosis (necroptosis) by activating TNFR1 receptors on their surface, followed by phosphorylation of Ripk1, Ripk3, and Mlkl. In turn, damage associated molecular patterns (DAMPs) released from necrotic cells activate pattern recognition receptors such as Tlr4 on the surface of glial cells, enhancing the inflammatory response. The expression of the genes highlighted in dark red is increased. (**B**) TNFR1 inactivation reduces NMDA excitotoxicity (**p-value < 0.01). (**C**) The expression of many genes involved in the inflammasome pathway is significantly increased in NMDA-treated retinas. The activity of this pathway leads to inflammation and regulated necrosis (pyroptosis). (**D**) Inactivation of one of the key genes, Gsdmd, in the inflammasome pathway increases the survival of RGCs in the NMDA-treated retinas (*p-value < 0.05). (**E**) Increased expression of iron signaling genes suggests ferrous (Fe^2+^) iron accumulation in NMDA-treated retinas. (**F**) Lowering iron levels using the oral iron chelator deferiprone (DFP) significantly increases RGC survival in the NMDA-treated retinas.

Regulated necrosis and inflammation are an inseparable couple that leads to significant tissue damage due to the positive feedback loop they form^16–20^. Often, the same signaling cascades that are responsible for inflammation also lead to cell regulated necrosis^16–20^. Among such signaling cascades is TNF signaling, the increased activity of which we found in the retinas 24 h after NMDA treatment (*Tnf*, log2FC = 3.47, padj = 5.2·10^–4^; *Tnfrsf1a* [TNFR1], log2FC = 1.41, padj = 5.7·10^–6^; *Ripk1*, log2FC = 1.57, padj = 2.1·10^–8^; *Ripk3*, log2FC = 3.17, padj = 6·10^–4^; *Mlkl*, log2FC = 3.01, padj = 1.6·10^–5^; etc.; Figs. 1G and 3A). This signaling cascade can stimulate an inflammatory response and activate a type of regulated necrosis known as necroptosis (Fig. 3A)^16–20^. The importance of TNF signaling in NMDA excitotoxicity is also evidenced by our GSEA analysis: the expression of many genes associated with biological processes and signaling cascades, such as GOBP Response to Tumor Necrosis Factor [FDR q-val = 0], GOBP Tumor Necrosis Factor Mediated Signaling Pathway [FDR q-val = 0.003], GOBP Necroptotic Process [FDR q-val = 0], and Reactome Ripk1 Mediated Regulated Necrosis [FDR q-val = 0.001], is increased (Fig. 2A). To investigate the contribution of the TNF signaling cascade to NMDA excitotoxicity, we used Tnf receptor 1 (TNFR1/*Tnfrsf1a*) knockout animals (TNFR1KO). It was previously shown that the Tnf cytokine exerts its toxic effect on RGCs by activating TNFR1 (Tnfrsf1a)^21–24^. To this end, TNFR1KO and WT animals were treated with NMDA (20 mM, 2 µL, left experimental eyes; n = 5) and PBS (2 µL, right control eyes; n = 5), as described above. The retinas of these animals were harvested 7 days later to determine the number of surviving GCL neurons and RGCs using Tubb3 and Rbpms markers (Supplementary Fig. S1). Our data indicate that the inactivation of the TNF signaling cascade results in significantly greater RGC survival compared to WT animals (Tubb3: 60 ± 7% [TNFR1KO] vs. 22 ± 4% [WT], *p-value* < *0.01*; Rbpms: 59 ± 8% [TNFR1KO] vs. 16 ± 3% [WT], *p-value* < *0.01*; Fig. 3B). Thus, TNF signaling-dependent inflammation and necroptosis contribute significantly to NMDA excitotoxicity.

Another example of a signaling cascade capable of leading to an inflammatory response and regulated necrosis is based on inflammasome activity and is dependent on gasdermin pore formation (Fig. 3C)^16–20^. Our data indicate increased expression of many genes of this signaling cascade in NMDA-treated retinas (*Nlrp3*, log2FC = 2.37, padj = 1.8·10^–5^; *Pycard* [ASC] log2FC = 1.93, padj = 5.2·10^–8^; *Casp1*, log2FC = 2.41, padj = 9.7·10^–6^; *Casp4*, log2FC = 2.88, padj = 3.5·10^–4^; *Il1b*, log2FC = 4.52, padj = 1.2·10^–10^; *Gsdmd*, log2FC = 1.37, padj = 5.7·10^–4^; etc.; Figs. 1G and 3C). The results of our GSEA analysis revealed the role of biological processes and signaling cascades such as GOBP Regulation of NLRP3 Inflammasome Complex Assembly [FDR q-val = 0.002], GOBP Pyroptosis [FDR q-val = 0.001], and Reactome Pyroptosis [FDR q-val = 0.001] in NMDA-treated retinas (Fig. 2A). All these data point to the contribution of inflammasome and a type of regulated necrosis known as pyroptosis to NMDA excitotoxicity. Since gasdermin pore formation is one of the key events leading to inflammation and pyroptosis, we used gasdermin D (*Gsdmd*) knockout (GsdmdKO) animals to assess the role of these processes. We chose this gene because it is the best studied relative to other members of the gasdermin family, and its expression was increased after treatment of retinas with NMDA (*Gsdmd*, log2FC = 1.37, padj = 5.7·10^–4^). To this end, GsdmdKO and WT animals were treated with NMDA (n = 6) and PBS (n = 6), as described above, and the retinas of these animals were collected after 7 days to study neuronal survival (Supplementary Fig. S1). We found that the *Gsdmd* inactivation leads to RGC survival compared to WT animals (Tubb3: 32 ± 2% [GsdmdKO] vs. 24 ± 2% [WT], *p-value* < *0.05*; Rbpms: 26 ± 2% [GsdmdKO] vs. 19 ± 2% [WT], *p-value* < *0.05*; Fig. 3D). However, the percentage of surviving RGCs was significantly lower compared to what we observed when TNFR1 was inactivated (Fig. 3B).

The results of our GSEA analysis suggest that free radicals and ferrous (Fe^2+^) iron as a catalyst for their production contribute to NMDA excitotoxicity. Evidence in favor of this hypothesis is the increased expression of genes such as *Steap3* (log2FC = 2.28, padj = 3.9·10^–14^), *Steap4* (log2FC = 1.59, padj = 1.5·10^–4^), *Ftl1* (log2FC = 1.37, padj = 1.9·10^–10^), and *Slc25a37* (Mfrn1; log2FC = 2.09, padj = 6.6·10^–8^) in NMDA treated retinas (Fig. 3E, Supplementary Data S1). Of note is the increased expression of *Steap3*: the enzyme it encodes is responsible for the generation of ferrous (Fe^2+^) iron from ferric (Fe^3+^) iron^25^. To evaluate the significance of these data, we used the oral iron chelator deferiprone (DFP). It has previously been shown that DFP significantly reduces the level of labile iron in the retina, protecting it from many pathologies^26–31^. We expected that lowering labile iron would lead to reduced NMDA excitotoxicity. To this end, we used two groups of animals: one was treated with DFP (1 mg/mL in drinking water; animals were given fresh DFP daily) and the other was not treated and served as a control (untr). Because a mouse drinks an average of 5 mL of water per day, our experimental mice consumed an average of 5 mg of DFP daily. Animals were pre-treated with DFP for 8 days prior to NMDA injection and were treated with DFP for 7 days after injection (Supplementary Fig. S1). After this period, the retinas of the experimental and control animals were collected and used to count the surviving GCL neurons and RGCs. We found that the quantity of RGCs in DFP–treated animals was significantly higher compared to untreated controls (Tubb3: 44 ± 4% [DFP] vs. 26 ± 2% [untr], *p-value* < *0.01*; Rbpms: 40 ± 4% [DFP] vs. 20 ± 2% [WT], *p-value* < *0.01*; Fig. 3F). Since high free radical and ferrous (Fe^2+^) iron levels lead to a type of regulated necrosis known as oxytosis/ferroptosis, our data suggest that oxytosis/ferroptosis contributes to NMDA excitotoxicity. Thus, putting together the results obtained here, it can be concluded that various types of regulated necrosis should contribute to NMDA excitotoxicity.

## Discussion

Overactivation of NMDA receptors makes a significant contribution to glutamate excitotoxicity and is known as NMDA excitotoxicity^8–11,13^. To study mechanisms of NMDA excitotoxicity in vivo, we used an animal model in which NMDA was injected into the vitreous of mice. We also used RNA-seq analysis, knockout animals, and animals treated with an iron chelator. The results of our RNA-seq analysis indicate activation of many signaling cascades involved in inflammation, programmed cell death, free radical production, oxidative stress, and iron and calcium metabolism 24 h after NMDA treatment. Meanwhile, the expression of genes whose activity is necessary to maintain normal neuronal function was reduced. Our data indicate an important role for the TNF signaling cascade and ferrous (Fe^2+^) iron production in retinal NMDA excitotoxicity. We found some neuroprotection upon inactivation of Gsdmd, whose activity leads to inflammasome-dependent inflammation and regulated necrosis. However, this neuroprotection was less pronounced compared to the neuroprotection that occurs when TNF signaling was inactivated or when ferrous (Fe^2+^) iron level was reduced.

NMDA excitotoxicity leads to death of RGCs and displaced amacrine cells in the ganglion cell layer and amacrine cells in the inner nuclear layer^8,9^. Significant necrosis of amacrine cells is observed already within the first hour after NMDA treatment^9^. Amacrine cell apoptosis can be detected only by the third hour after NMDA treatment^8,9^. At the same time, published data and our results presented here indicate that RGCs are more resistant than amacrine cells to NMDA excitotoxicity. The in vitro results obtained in Dr. Barres’ laboratory indicate that the presence of glutamate or NMDA in the cell culture medium either does not lead to the RGC death, or it even promotes their survival^9,12^. At the same time, the results of many investigators, including our data, indicate a significant loss of RGCs after NMDA treatment in vivo^2–6,8,10,11,13^. However, the RGCs began to die much later than amacrine cells, that is, their death was delayed^8–11^. How could this contradiction be explained? It is an established fact that cell death via necrosis leads to a significant inflammatory response in the tissue^16–20^. Many damage-associated molecular patterns (DAMPs: e.g., Hsp70 and Hmgb1) released from necrotic cells activate the same pattern recognition receptors (Tlr4 as an example) as products of pathogens, resulting in a strong inflammatory response^16–20,32–34^. The secretion of the Tnf cytokine is one of the critical events in the inflammatory response^35^. Our RNA-seq data indicate a significant inflammatory response in the retina 24 h after NMDA treatment. Necrosis of amacrine cells in the first hours after NMDA treatment could explain the occurrence of such a strong inflammatory response. Our data also provide evidence of activation of the TNF signaling cascade 24 h after NMDA treatment. Meanwhile, inactivation of this cascade leads to significant RGC survival in NMDA treated retinas. Our data and the results of other investigators indicate that there is significant RGC death in the presence of the Tnf cytokine in vivo and in vitro^8,22–24,31,36–39^. It has also been shown that inhibition of this cytokine leads to significant RGC survival after NMDA treatment^8^. The totality of these data allows us to propose the following mechanism. Necrosis of amacrine cells in the first hours after NMDA treatment leads to an inflammatory response, including Tnf production and secretion. In turn, high Tnf levels lead to the death of RGCs, which could explain their delayed death. It should be noted that Tnf leads to cell death not only via apoptosis, but also via regulated necrosis known as necroptosis^35^. Our RNA-seq data indicate an increased expression of *Ripk1*, *Ripk3*, and *Mlkl* genes that trigger necroptosis^35^. Thus, RGC and amacrine cell regulated necrosis could lead to increased inflammatory reaction by launching a positive feedback loop in which regulated necrosis promotes inflammation and inflammation triggers regulated necrosis. All this together can result in significant RGC death in NMDA treated retinas.

The results of our study indicate that free radicals (ROS/RNS) and ferrous (Fe^2+^) iron as a catalyst for their production contribute to NMDA excitotoxicity. By lowering the level of labile iron in the NMDA-treated retinas using the oral iron chelator DFP, we were able to achieve significant RGC survival. Increased levels of free radicals in the NMDA-treated retinas and their negative impact on RGC survival have been shown previously^3–6,40,41^. However, the contribution of ferrous (Fe^2+^) iron to retinal NMDA excitotoxicity has been examined in only one study by Sakamoto et al. to the best of our knowledge^42^. In agreement with our data, these authors showed increased labile ferrous (Fe^2+^) iron, free radical, and oxidative stress levels in NMDA-treated retinas^42^. At the same time, the authors showed a decrease in labile ferrous (Fe^2+^) iron and oxidative stress levels and an increase in the level of surviving RGCs in the NMDA- and iron chelator-treated retinas^42^. These findings suggest an important role for ferrous (Fe^2+^) iron as a catalyst for free radical production in retinal NMDA excitotoxicity. These data also show that since oxytosis/ferroptosis depends on high ferrous (Fe^2+^) iron and free radical levels, this type of regulated necrosis should be involved in NMDA excitotoxicity^43,44^. Thus, oxytosis/ferroptosis, together with necroptosis, would contribute to maintaining the positive feedback loop described in the previous paragraph leading to significant RGC death in the NMDA-treated retinas.

Inflammasome assembly is required to activate caspase-1, which cleaves pro-Il1b to generate the mature cytokine and cleaves gasdermins (GSDM) to generate pore-forming fragments that, in turn, targets the membrane and allows the release of mature Il1b^18,45^. However, the presence of a large number of GSDM pores on the cell membrane over a long period of time can lead to cell death via regulated necrosis, known as pyroptosis^18,45^. Thus, inflammasome assembly can lead to both inflammation and pyroptosis in the tissue. The results of our GSEA analysis suggested a contribution of inflammasome activity and pyroptosis to retinal NMDA excitotoxicity. Since GSDM pore formation is one of the key events leading to the inflammatory response and pyroptosis, we used Gsdmd deficient (GsdmdKO) animals to evaluate the role of these processes in NMDA excitotoxicity. While the percentage of surviving RGCs was higher in GsdmdKO mice compared to WT controls, this value was significantly lower than the values obtained in TNFR1KO animals or in iron chelator-treated animals (Fig. 3). These data suggest that the inflammasome role is probably less significant than the role of TNF signaling and ferrous (Fe^2+^) iron in retinal NMDA excitotoxicity. Tsoka et al. came to a similar conclusion when they examined the contribution of inflammasome-mediated inflammation to retinal NMDA excitotoxicity^46^.

**Figure 4:**
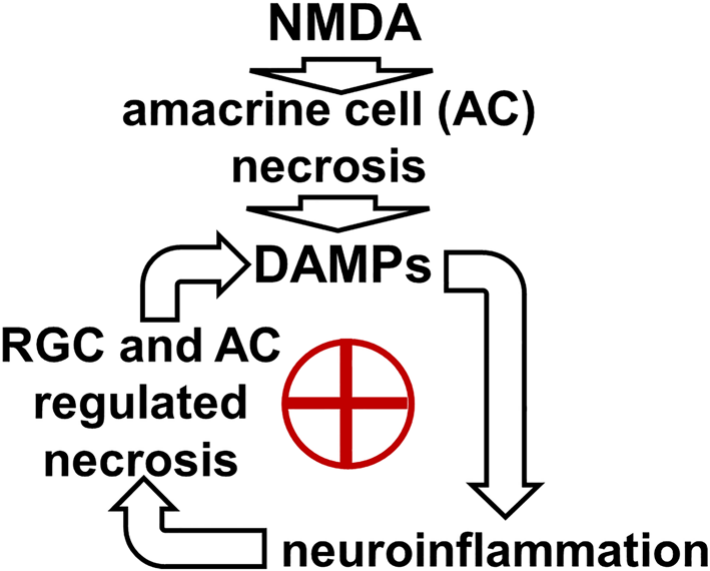
This figure illustrates our proposed mechanism for retinal NMDA excitotoxicity. According to this mechanism, NMDA-mediated amacrine cell (AC) death via necrosis triggers a positive feedback loop, leading to significant RGC death and retinal degeneration.

In conclusion, the results of our study and previously published data support a mechanism of retinal NMDA excitotoxicity in which overactivation of NMDA receptors leads to rapid death of amacrine cells via necrosis (Fig. 4). In turn, DAMPs released from necrotic amacrine cells trigger a strong inflammatory response, including the activation of TNF signaling. The Tnf cytokine is toxic to RGCs and, thus, high Tnf levels lead to RGC death through apoptosis and necroptosis (one of the types of regulated necrosis). This mechanism can explain the delayed death of RGCs in retinal NMDA excitotoxicity. We do not rule out that Tnf may also lead to the death of remaining amacrine cells through apoptosis and necroptosis. Our results and previously published data also suggest that ferrous (Fe^2+^) iron-dependent regulated necrosis (oxytosis/ferroptosis) of RGCs and amacrine cells contributes to NMDA excitotoxicity. In turn, RGC and amacrine cell necroptosis and oxytosis/ferroptosis should promote inflammation and retinal damage by launching the positive feedback loop in which regulated necrosis promotes inflammation, which subsequently triggers regulated necrosis (Fig. 4). The proposed mechanism provides insight into retinal NMDA excitotoxicity. Since NMDA excitotoxicity is part of glutamate excitotoxicity, which, in turn, is an important contributor to many retinal diseases, our proposed mechanism highlights several targets for drug development that could help patients suffering from such retinal diseases.

## Methods

### Animals and ethics statement

All procedures were executed in compliance with the National Institutes of Health (NIH) Guide for the Care and Use of Laboratory Animals and according to the University of Miami Institutional Animal Care and Use Committee (IACUC) approved protocol (Protocol #: 21-070). TNFR1 and Gsdmd knockout animals (TNFR1KO and GsdmdKO, respectively; these knockouts have the C57BL/6 J genetic background) and C57BL/6J mice as the wild-type (WT) controls were received from the Jackson Laboratory (Bar Harbor, ME, USA; stock numbers 003242, 032663, and 000664). The oral iron chelator deferiprone (DFP, 1 mg/mL, #379409, MilliporeSigma, St. Louis, MO, USA) was delivered to animals via drinking water. We used 2–4-month-old male and female mice to address sex as a biological variable. Animals were housed under standard conditions of humidity and temperature, were given free access to food and water, and had a 12-h light to dark cycle. All methods were completed and reported in accordance with ARRIVE guidelines.

### Animal model of NMDA excitotoxicity

The animals were anaesthetized by intraperitoneal injection of ketamine (80 mg/kg)/xylazine (10 mg/kg) to perform intravitreal injections. We used change in heart rate in response to tail pinch and corneal reflex as an indication of the level of anesthesia. Intravitreal injections were performed under a microsurgical microscope using glass pipettes with a diameter of approximately 150 µm at the tip. Each eye was punctured at the upper nasal limbus and a volume of 2 µL of NMDA (20 mM, M3262, MilliporeSigma, St. Louis, MO, USA) or phosphate-buffered saline (PBS, #10010031, ThermoFisher Scientific, Waltham, MA, USA) was injected into the vitreous of mice. To allow diffusion of the solutions the pipette was kept in place for about 15 s. To collect retinas, mice were euthanized in accordance with the recommendations of the Panel on Euthanasia of the American Veterinary Medical Association (AVMA). Briefly, the animal is placed in a plastic, top-opening Plexiglass cage, which is covered with a lid. The flow of CO_2_ from a cylinder is applied for a few minutes to establish a high concentration of CO_2_ at the bottom of the cage. After breathing has stopped and the mouse is unconscious, euthanasia is completed by cervical dislocation (a secondary physical method). In this simple and humane method of killing small rodents, the animal is held by its tail, placed on a flat surface, and stretched out so that a pencil or similar object can be placed across the back of its neck. A firm and quick pull on the base of the tail dislocates the neck, killing the animal instantly.

### RNA purification and quality control

Total RNA was purified from experimental and control retinas using RNeasy Plus Mini Kit (#74134, Qiagen, Hilden, Germany) as described previously^47^. RNA quantity and quality was measured by Qubit 4 Fluorometer and the NanoDrop One spectrophotometer (ThermoFisher Scientific, Waltham, MA, USA). 2100 Bioanalyzer Instrument (Agilent Technologies, Santa Clara, CA, USA) was used to assess RNA integrity. The RNA samples, which had a RIN score of 8 or higher, were used to prepare RNA-seq libraries.

### RNA-seq library preparation and sequencing

To prepare RNA-seq libraries, we used Illumina Stranded mRNA Prep Kit (#20040532, Illumina, San Diego, CA, USA) and IDT® for Illumina® RNA UD Indexes Set A (#20040553, Illumina, San Diego, CA, USA) according to manufacturer's instructions. The quantity and quality of the RNA-seq libraries were measured using Qubit 4 Fluorometer, NanoDrop One spectrophotometer, and 2100 Bioanalyzer Instrument. The RNA-seq libraries were multiplexed and then sequenced from both ends on the Illumina Novaseq 6000 with a 2 × 150 paired end (PE) configuration. The next-generation sequencing (NGS) was carried out in the Advanced Genomics Core at the University of Michigan. The FASTQ files obtained in this study were uploaded to the BioProject database (https://www.ncbi.nlm.nih.gov/bioproject/) and are available under the accession number PRJNA1015283.

### RNA-seq data analysis

We used STAR RNA-seq aligner and a basic workflow to align paired-end reads^48^. The HTseq package was used to determine how many reads overlap each of the mouse genes^49^. The differential gene expression analysis was conducted using the DESeq2 Bioconductor package^50^. The ViDGER (visualization of differential gene expression results using R) Bioconductor package was used for visualizations of our RNA-seq data. We used Gene Set Enrichment Analysis (GSEA, a powerful analytical method for interpreting RNA-seq data) to identify important signaling cascades and biological processes^15^.

### Immunohistochemistry of flat-mounted retinas and counting neurons in the ganglion cell layer

Experimental and control eyes of mice were enucleated, fixed with 4% paraformaldehyde (PFA) in phosphate-buffered saline (PBS, pH 7.4) for 1 h and then transferred to PBS. The retinas were removed, washed with PBS, permeabilized with 0.5% Triton X-100 in PBS for 1 h, blocked with 0.5% Triton X-100 containing 10% donkey (or goat) serum in PBS for 1 h, and then incubated overnight in 0.2% Triton X-100/10% donkey (or goat) serum in PBS containing Tubb3 antibody (1:250; 802001, BioLegend, San Diego, CA, USA) and Rbpms antibody (1:400, GTX118619, GeneTex, Irvine, CA, USA). The next day, the retinas were washed with PBS, and incubated with species-specific secondary fluorescent antibodies (ThermoFisher Scientific, Waltham, MA, USA). Negative controls were incubated without primary antibodies. Imaging was performed with Leica STELLARIS confocal microscope (Leica Microsystems, USA). Tubb3- and Rbpms-positive neurons in the ganglion cell layer (GCL) were imaged randomly at 20 × magnification to collect images from four retinal quadrants at the same eccentricity from the optic disc in the central retina (one image per quadrant, total 4 images), middle retina (two images per quadrant, total 8 images), and peripheral retina (two images per quadrant, total 8 images). A total of 20 images were collected on each experimental and control retina. Each image includes the retinal area of 342 µm × 342 µm. Examples of such images are shown in Fig. 3. The numbers of Tubb3- and Rbpms-positive neurons were counted with ImageJ software (https://imagej.nih.gov/). Neuronal survival in NMDA-treated retinas was calculated as a percentage of the mean cell number in fellow control retinas.

### Statistical analysis

We used the unpaired Student’s t-test for experiments containing one variable. *P-values* equal to or less than 0.05 were considered statistically significant. Protocols using a range of genotypes or drug treatments were designed with individual treatments being assigned in a random fashion. Treatments were assigned blindly to the experimenter by another individual in the laboratory. Generation and analysis of next-generation sequencing (NGS) data were carried out in-house according to ENCODE standards and pipelines with n = 4 for RNA-seq data.

### Supplementary Information

Supplementary Information 1.

Supplementary Information 2.

Supplementary Figure S1.

## Data Availability

The datasets generated and analyzed during the current study are available in the BioProject database (accession number PRJNA1015283) and in the article/Supplementary Data.
