# Supplementary material for: Molecular mechanisms of NMDA excitotoxicity in the retina
Source: Sci Rep. 2023 Oct 27;13:18471. doi: 10.1038/s41598-023-45855-0 (PMC10611720; doi:10.1038/s41598-023-45855-0)
Supplement: Supplementary file 3 — Supplementary Figure S1. [file 41598_2023_45855_MOESM3_ESM.pdf]

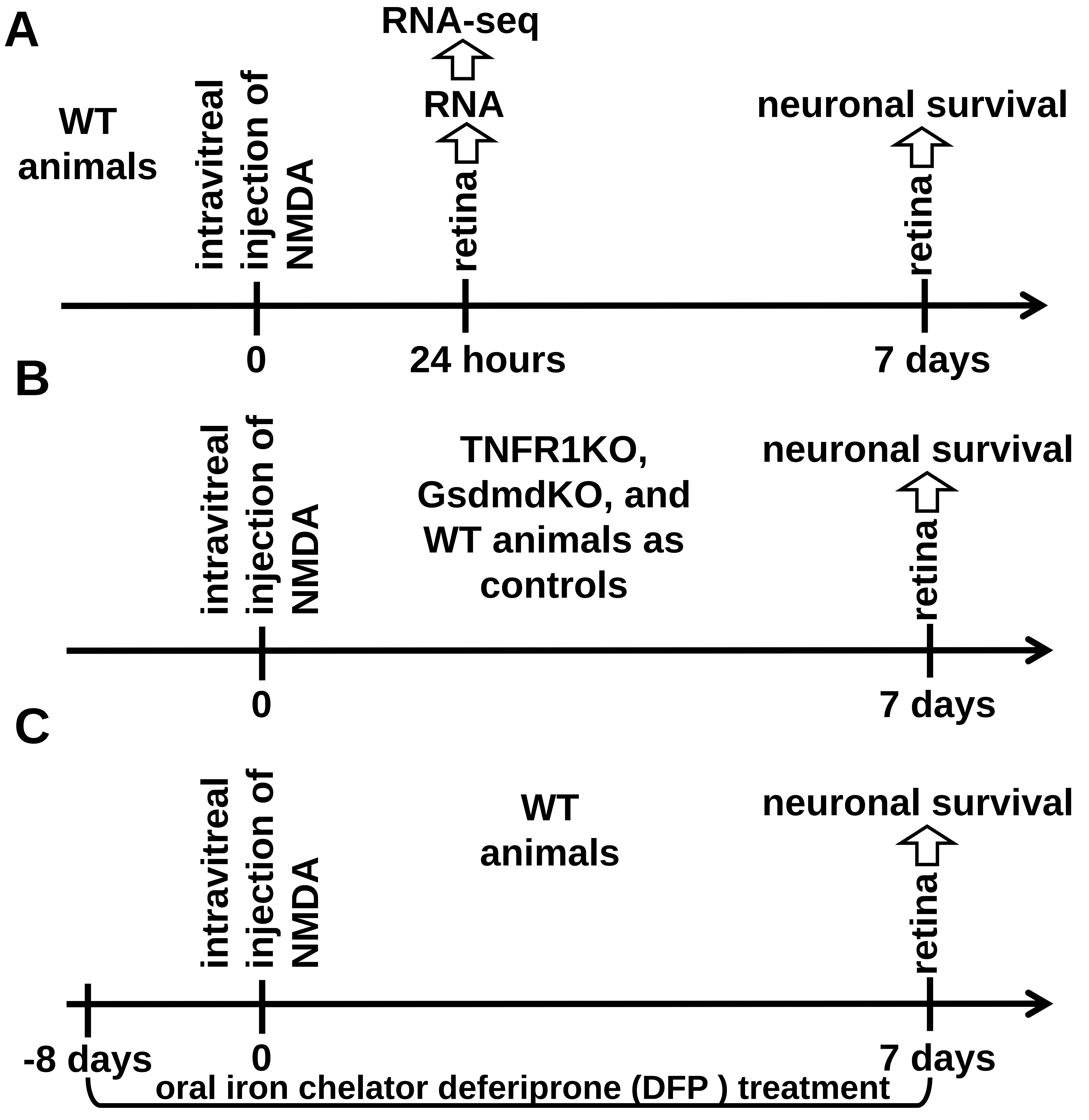


**Supplementary Figure S1:** The timeline of experimental procedures. **A)** To identify signaling cascades whose activation leads to NMDA excitotoxicity, we collected retinas 24 hours after NMDA treatment and used them in RNA-seq analysis. The percentage of surviving GCL neurons was determined 7 days after treatment. **(B, C)** To investigate the role of signaling cascades whose activity was increased after NMDA treatment, we used knockout animals, animals treated with the oral iron chelator DFP, and WT animals as controls. In this case, retinas of these animals were collected 7 days after NMDA treatment and used to determine the number of surviving GCL neurons. It should be noted that WT animals were pre-treated with DFP for 8 days prior to NMDA injection and were treated with DFP for 7 days after injection.
